# Neural encoding of voice pitch and formant structure at birth as revealed by frequency-following responses

**DOI:** 10.1038/s41598-021-85799-x

**Published:** 2021-03-23

**Authors:** Sonia Arenillas-Alcón, Jordi Costa-Faidella, Teresa Ribas-Prats, María Dolores Gómez-Roig, Carles Escera

**Affiliations:** 1 Brainlab-Cognitive Neuroscience Research Group, Department of Clinical Psychology and Psychobiology, University of Barcelona, P. Vall d’Hebron 171, 08035 Barcelona, Catalonia Spain; 2 Institute of Neurosciences, University of Barcelona, Barcelona, Catalonia Spain; 3 Institut de Recerca Sant Joan de Déu, Esplugues de Llobregat, Catalonia Spain; 4 BCNatal-Barcelona Center for Maternal Fetal and Neonatal Medicine (Hospital Sant Joan de Déu and Hospital Clínic), University of Barcelona, Barcelona, Catalonia Spain

**Keywords:** Cognitive neuroscience, Language, Paediatric research, Neuroscience, Paediatrics, Neonatology, Paediatric research, Biomarkers, Predictive markers

## Abstract

Detailed neural encoding of voice pitch and formant structure plays a crucial role in speech perception, and is of key importance for an appropriate acquisition of the phonetic repertoire in infants since birth. However, the extent to what newborns are capable of extracting pitch and formant structure information from the temporal envelope and the temporal fine structure of speech sounds, respectively, remains unclear. Here, we recorded the frequency-following response (FFR) elicited by a novel two-vowel, rising-pitch-ending stimulus to simultaneously characterize voice pitch and formant structure encoding accuracy in a sample of neonates and adults. Data revealed that newborns tracked changes in voice pitch reliably and no differently than adults, but exhibited weaker signatures of formant structure encoding, particularly at higher formant frequency ranges. Thus, our results indicate a well-developed encoding of voice pitch at birth, while formant structure representation is maturing in a frequency-dependent manner. Furthermore, we demonstrate the feasibility to assess voice pitch and formant structure encoding within clinical evaluation times in a hospital setting, and suggest the possibility to use this novel stimulus as a tool for longitudinal developmental studies of the auditory system.

## Introduction

Spoken language is arguably the most prevalent form of human communication. Experimental evidence suggests a universal organic basis for language acquisition, based on the identical development of speech perception pathways observed across different populations, languages and cultures^1–3^. Speech perceptual skills have been well characterized along the lifespan, especially with regard to their maturation during the first year^4–6^. However, less is known about their functional state during the very first hours after birth, when humans newly encounter the rich and challenging complexity of the external acoustic environment. A highly efficient auditory system becomes hence a requisite for proper language acquisition, as the complex and dynamic acoustic signal of speech conveys only very slight spectral and temporal cues for speech sound discrimination^7^.

Previous studies have shown that the auditory system of newborns and infants is able to handle several aspects related to pitch processing, such as tracking pitch contours^8–15^, higher-order frequency direction relationships^16^, processing a missing fundamental^17^ or exhibiting relative pitch by discriminating transposed melodies^18,19^. Likewise, the newborn auditory system appears able to discriminate phonemes^20–22^ even when only based upon vowel formant structure changes or duration^20,23,24^. And yet, the low level neural underpinning of these abilities remains to be established.

Using non-invasive electroencephalography recordings, auditory brainstem responses (ABR) evoked to acoustic transients, such as click stimuli, have successfully been used to assess the integrity of the auditory pathway^25–27^. However, periodic acoustic stimuli also elicit a particular brain response of subcortical and cortical origin, known as the frequency-following response (FFR)^28–30^. The FFR reflects with high fidelity the encoding of periodic temporal envelope modulations (FFR_ENV_) and temporal fine structure harmonic constituents (FFR_TFS_) of a stimulus (Aiken and Picton^31^ following the terminology proposed by Krizman and Kraus^29^). In language studies, these two components of the FFR have been respectively regarded as indexes of two perceptual properties of speech sounds: voice pitch contour and formant structure^29,31^.

FFR recordings are increasingly considered a valuable tool to index the current functional state of the auditory system and to predict the future development of language^32^, since disruptions in the FFR elicited by speech sounds relate to deficits in phonological awareness, reading impairments and dyslexia^33–36^. The potential of the FFR as a biomarker for auditory deficits and their relation to literacy skills has thus been proposed^11,15,37–41^. However, most developmental studies on the FFR targeted babies of several months of age (e.g.,^42–44^), toddlers, infants or years-old children (e.g.,^37,40,44–49^), with only a few published reports on newborns^8–13,50^. Thus, knowledge about the expected speech perceptual skills in newborns, who are more vulnerable than older age groups to hearing damage^3,15^, may aid the early detection of language impairments and guide appropriate interventions benefitting from the massive neural plasticity during the first years of life^47,51–54^.

Moreover, while newborn studies have focused on the assessment and maturation of pitch processing through the analysis of the FFR_ENV_, to date, none addressed formant structure encoding in a systematic manner^9,11,15^. To the best of our knowledge, only a recent study from our lab^11^, providing a normative newborn database of FFR_ENV_ properties, attempted, as a secondary aim, to reveal whether the neonate auditory system was able to discriminate sounds differing in their fine structure (/da/ vs. /ga/). While the results were negative, the apparent lack of formant structure encoding may be due to several reasons: (a) the short duration of the consonant transition (47 ms) and its formant change, which limit the resolution of the computed spectral information^42^; (b) the high frequency content of the stimuli (/da/ F_2_ = 1438–1214 Hz, /ga/ F_2_ = 1801–1214 Hz), which elicits diminished FFR amplitudes that are difficult to recognize^55^; (c) the fact that phase-locking to higher frequencies develops later than to lower ones^39,42^; and, ultimately, (d) the nature of the analyzed signal (FFR_ENV_), which emphasizes temporal envelope information representation at the expense of temporal fine structure^31^. Thus, it still remains unclear whether newborns cannot yet precisely track formant changes in complex sounds or if stimulation parameters used so far were not suited to reveal this ability.

Furthermore, in newborn FFR research, time is of the essence. Recording time constraints determine what stimulus encoding abilities can be studied, and it is even a more challenging issue when newborn research is conducted in a hospital setting. In addition to the ease of waking up or disturbing sleep, the hospital environment requires frequent and continuous access to the baby and the mother for routine tests to discard serious health issues, interventions, in-depth health evaluations, and any other kind of neonatal care. Taking this into account, it would be unsuitable to carry out recording sessions lasting between 40^23^ and 50^8^ min, typical of speech-sound discrimination studies. The most adequate session duration would be between 20 and 30 min^40,42^. Therefore, devising a new single stimulus that would allow a proper assessment of both the FFR_ENV_ and FFR_TFS_ simultaneously within recording times compatible with infant research is required. This would provide a snapshot of the functional state of speech sound processing mechanisms at birth and, ultimately, help better understand how the encoding of this complex auditory signal matures.

Thus, the aim of the present study was to characterize the functional maturity of voice pitch contour and formant structure encoding mechanisms in the newborn population with non-invasive electrophysiological recordings, using the adult population as a reference. To that end, we developed a novel speech stimulus that allows the simultaneous assessment of both components of the speech signal through analyzing the FFR_ENV_ and the FFR_TFS_, and which is at the same time compatible with clinical evaluation time constrains in a hospital setting.

FFRs were recorded to a novel two-vowel (/oa/) speech stimulus with a rising pitch ending. In order to estimate voice pitch encoding from the temporal envelope of the recorded neural response, we computed the FFR_ENV_ and analyzed it (spectral measures at the fundamental frequency [F_0_] peak; pitch tracking measures extracted from stimulus-to-response cross-correlations and signal autocorrelations) accounting for the different steady and rising pitch sections of the stimulus (both during the /a/ vowel). In order to estimate formant structure encoding from the temporal fine structure of the recorded response, we computed the FFR_TFS_ and analyzed it (spectral measures at the first formant [F_1_] frequency peak) accounting for the different /o/ and /a/ vowel sections of the stimulus (both during the steady pitch section)^8–13,29,42,46,56^.

Given that the human auditory system is able to encode changes in voice pitch with great precision starting from the first days of life^9^, we would expect no significant differences between newborns and adults in spectral amplitudes at F_0_ or in pitch tracking accuracy measures computed from the FFR_ENV_. Because the capacity of neurons in the auditory system to phase-lock their activity to higher sound frequencies develops later than to lower ones and continues improving during the first year of life^39,42^, we would expect newborns to exhibit overall smaller FFR_TFS_ spectral amplitudes at F_1_ peak frequencies than adults. However, we had no clear hypotheses as to whether the newborn FFR_TFS_ would reflect a discriminative encoding of vowel formant structure as it does in adults^57^ and, if so, whether that discriminative encoding would depend on F_1_ center frequency. The evidence suggesting that the newborn auditory system discriminates vowel changes^20–24^, is based upon recordings of event-related potentials (ERPs) reflecting higher-order auditory system computations. But, the fundamentally different nature of the FFR as a phase-locked neural response reflecting the acoustic waveform with high precision, and the fact that this low-level encoding of acoustic features is immature at birth, at least for higher frequencies, precluded us at this point to have strong hypotheses on the newborn’s auditory system’s capabilities.

## Results

**Figure 1:**
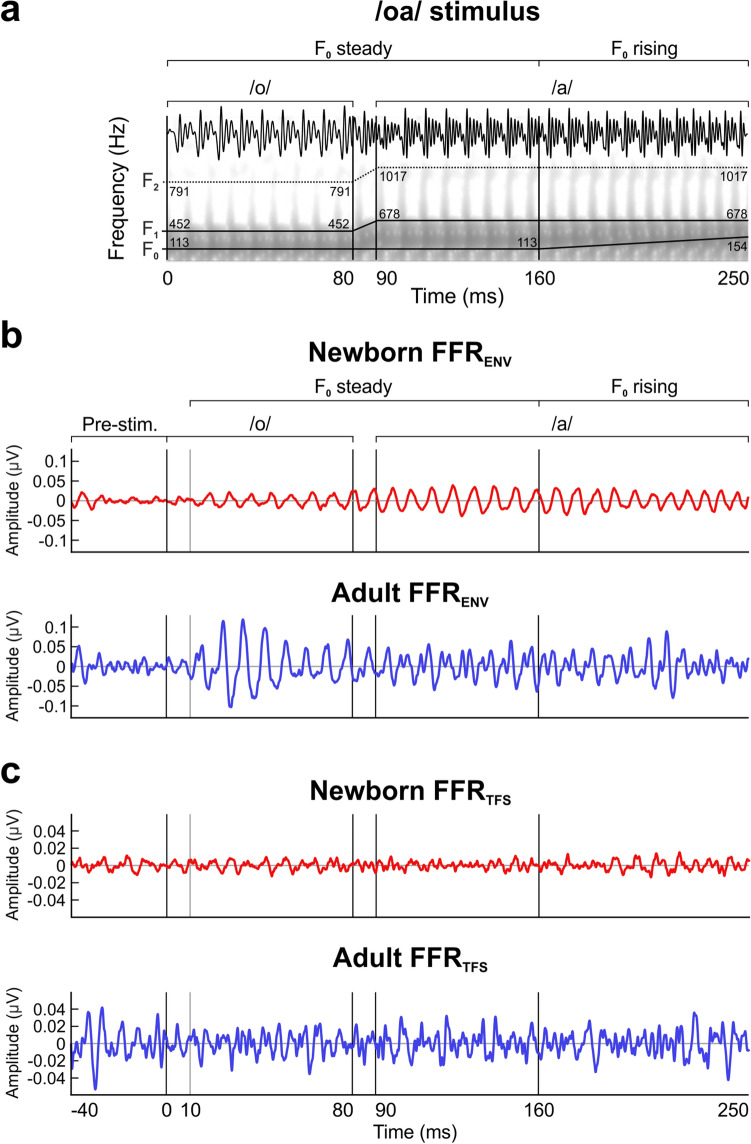
Temporal representation of the stimulus (**a**); FFR_ENV_ (**b**) and FFR_TFS_ (**c**). (**a**) Time waveform (top) and spectrogram of the /oa/ stimulus with schematic overlay of the formant structure trajectory (targeted F_0_ and F_1_ in solid lines; non-analyzed F_2_ depicted in dotted line). (**b**) Grand averaged time-domain waveform of the FFR_ENV_ from newborns (top red) and adults (bottom blue), obtained by averaging the neural responses to the two stimulus polarities. (**c**) Grand averaged time-domain waveform of the FFR_TFS_ from newborns (top red) and adults (bottom blue), obtained by subtracting the neural responses to the two stimulus polarities.

Temporal envelope-following responses (FFR_ENV_) and temporal fine structure-following responses (FFR_TFS_) elicited by a two-vowel syllable, rising-pitch ending, /oa/ stimulus (Fig. 1a) were collected from 34 newborns and 18 adult participants. In order to assess voice pitch contour and formant structure encoding in depth, neural responses were analyzed according to the sound features of the different stimulus sections. Below, we provide descriptive statistics and comparisons for a comprehensive number of parameters extracted from the FFR_ENV_ and FFR_TFS_ (see “Methods” for a detailed description). Statistically non-significant results can be found in Suppl. Table 1.

**Table 1:** Descriptive statistics for FFR_ENV_ derived parameters: neural lag; F_0_ spectral amplitude and SNR computed for the steady pitch section; stimulus-to-response cross-correlation, pitch error and pitch strength computed separately for each section of the stimulus (/a/ steady section; /a/ rising section).

Measure	Mean	SD	Median	Q_1_	Q_3_	IQR	Minimum	Maximum
**Neural lag (ms; from 10 to 250 ms)**								
Newborns	9.33	1.74	9.26	8.70	10.01	1.31	3.53	12.60
Adults	6.26	1.21	5.85	5.55	6.47	0.92	5.10	9.75
**F**_**0**_** spectral amplitude (µV; from 10 to 160 ms)**								
Newborns	0.01	0.01	0.01	< 0.01	0.02	0.01	< 0.01	0.03
Adults	0.02	0.01	0.02	0.01	0.02	0.01	0.01	0.04
**F**_**0**_** SNR (from 10 to 160 ms)**								
Newborns	4.36	4.72	5.86	0.82	7.83	7.01	−11.07	10.95
Adults	4.53	3.43	4.70	3.01	7.26	4.25	−3.52	9.40
**/a/ steady section **(90–160 ms)								
*Cross-correlation (Pearson’s r)*								
Newborns	0.18	0.06	0.18	0.12	0.23	0.11	0.06	0.30
Adults	0.20	0.05	0.20	0.17	0.23	0.07	0.07	0.27
*Pitch error (Hz)*								
Newborns	11.66	7.15	10.18	5.64	16.55	10.90	2.76	28.73
Adults	9.45	3.40	9.77	6.45	12.07	5.63	3.27	15.29
*Pitch strength (r)*								
Newborns	0.60	0.18	0.57	0.45	0.74	0.29	0.32	0.88
Adults	0.55	0.09	0.55	0.46	0.62	0.15	0.43	0.76
**/a/ rising section (160–250 ms)**								
*Cross-correlation (Pearson’s r)*								
Newborns	0.11	0.04	0.10	0.08	0.13	0.05	0.05	0.18
Adults	0.10	0.02	0.10	0.09	0.12	0.03	0.06	0.15
*Pitch error (Hz)*								
Newborns	11.72	7.08	10.44	5.50	16.25	10.75	2.60	28.15
Adults	9.50	3.34	9.87	6.55	12.00	5.45	3.13	15.03
*Pitch strength (r)*								
Newborns	0.60	0.18	0.56	0.45	0.74	0.29	0.32	0.88
Adults	0.55	0.09	0.55	0.46	0.61	0.15	0.44	0.75

*SD* standard deviation, *Q*_*1*_ first quartile (25th percentile), *Q*_*3*_ third quartile (75th percentile), *IQR* interquartile range.

### Neural transmission delay

#### Neural lag

Newborns showed a significantly longer neural lag (an estimation of FFR latency) compared to adults (U_(50)_ = 59, *p* < 0.001, Cohen’s *d* = 0.659). Descriptive statistics can be found in Table 1.

### Assessment of voice pitch encoding from FFR_ENV_

In order to determine the strength of the representation of the F_0_ and assess the accuracy in tracking F_0_ changes, our /oa/ stimulus was devised to feature a steady pitch during its initial section (113 Hz; 0–160 ms) and a linearly increasing pitch during its final section (113–154 Hz; 160–250 ms) (Fig. 1a). To accentuate the FFR components corresponding to the encoding of the stimulus envelope (mainly the F_0_) while suppressing those related to the fine structure, thus controlling for vowel changes that occur along the different sections of the stimulus, we computed the FFR_ENV_. Grand-average FFR_ENV_ waveforms are shown in Fig. 1b for both groups separately (newborns and adults). All descriptive statistics for FFR_ENV_ derived parameters can be found in Table 1.

#### Spectral amplitude at F_0_ peak

**Figure 2:**
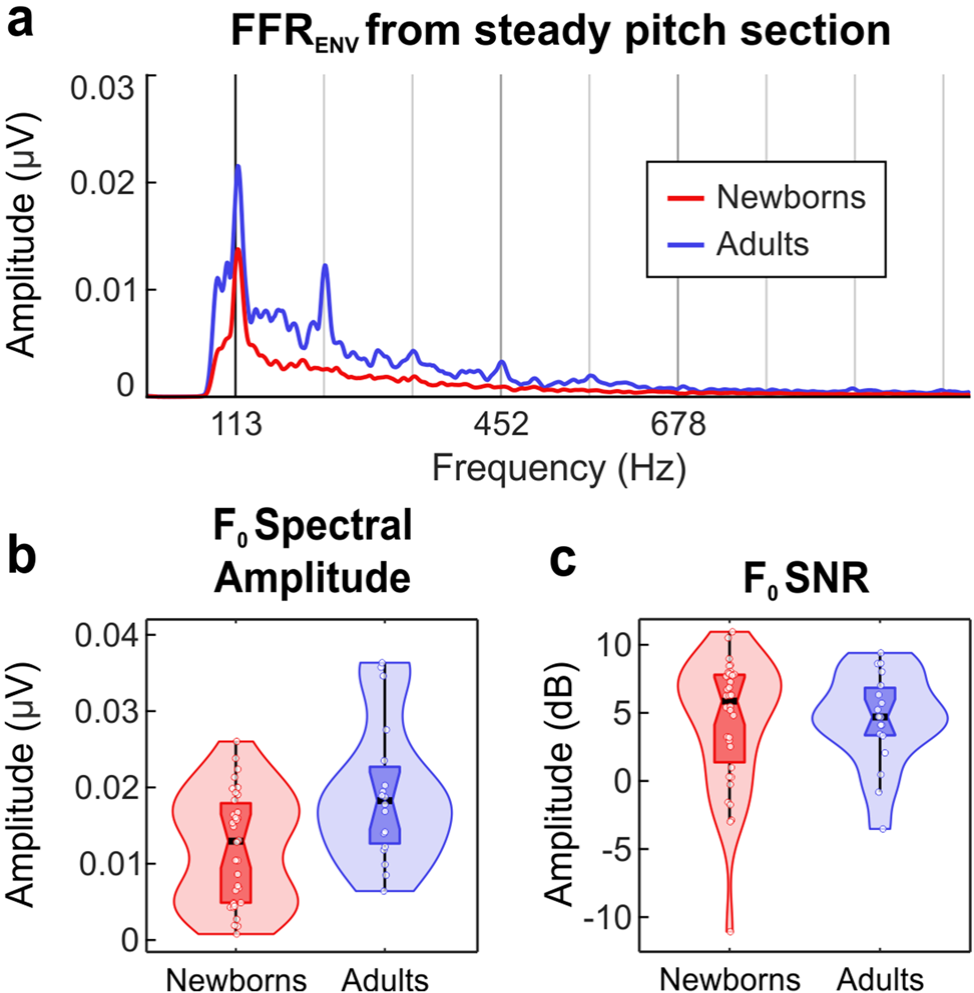
Amplitude FFR_ENV_ spectra (**a**) and data distributions (violin plots) of F_0_ spectral amplitude (**b**) and F_0_ SNR (**c**) parameters extracted from the steady pitch section of the stimulus, by averaging the neural responses to the two stimulus polarities from both groups separately. Scatter plots show all tested participants in each group. In each plot, horizontal black line and vertical black line indicate the median and the interquartile range, respectively.

The spectral amplitude at F_0_ peak (113 Hz) during the steady pitch section of the stimulus (10–160 ms) was calculated as an indicator of the magnitude of neural phase-locking at that specific frequency^49^. Newborns exhibited significantly reduced spectral amplitudes at F_0_ peak as compared to adults (t_(50)_ = − 3.079, *p* = 0.003, Cohen’s *d* = − 0.831). The corresponding amplitude spectra in the frequency domain computed along the steady pitch stimulus section is shown in Fig. 2a. Figure 2b illustrates the distribution of F_0_ spectral amplitude values obtained for each group.

#### Signal-to-noise ratio

The signal-to-noise ratio (SNR) at F_0_ peak during the steady pitch section of the stimulus was taken as an estimation of the relative spectral magnitude of the response. No significant group differences were found. Figure 2c illustrates the distribution of F_0_ SNR values obtained per group.

#### Stimulus-to-response cross-correlation

The stimulus-to-response cross-correlation was taken as a measure of the accuracy with which the FFR_ENV_ reproduced the stimulus waveform, separately for the /a/ steady and /a/ rising pitch contour stimulus sections. Lower stimulus-to-response cross-correlation values were obtained during the rising pitch section (mean ± SD; /a/ rising = 0.11 ± 0.03) as compared to the steady pitch section (mean ± SD; /a/ steady = 0.18 ± 0.06) (Z = − 5.774, *p* < 0.001, Cohen’s *d* = 0.801). No significant group differences or group per stimulus section interaction were found.

#### Pitch error

**Figure 3:**
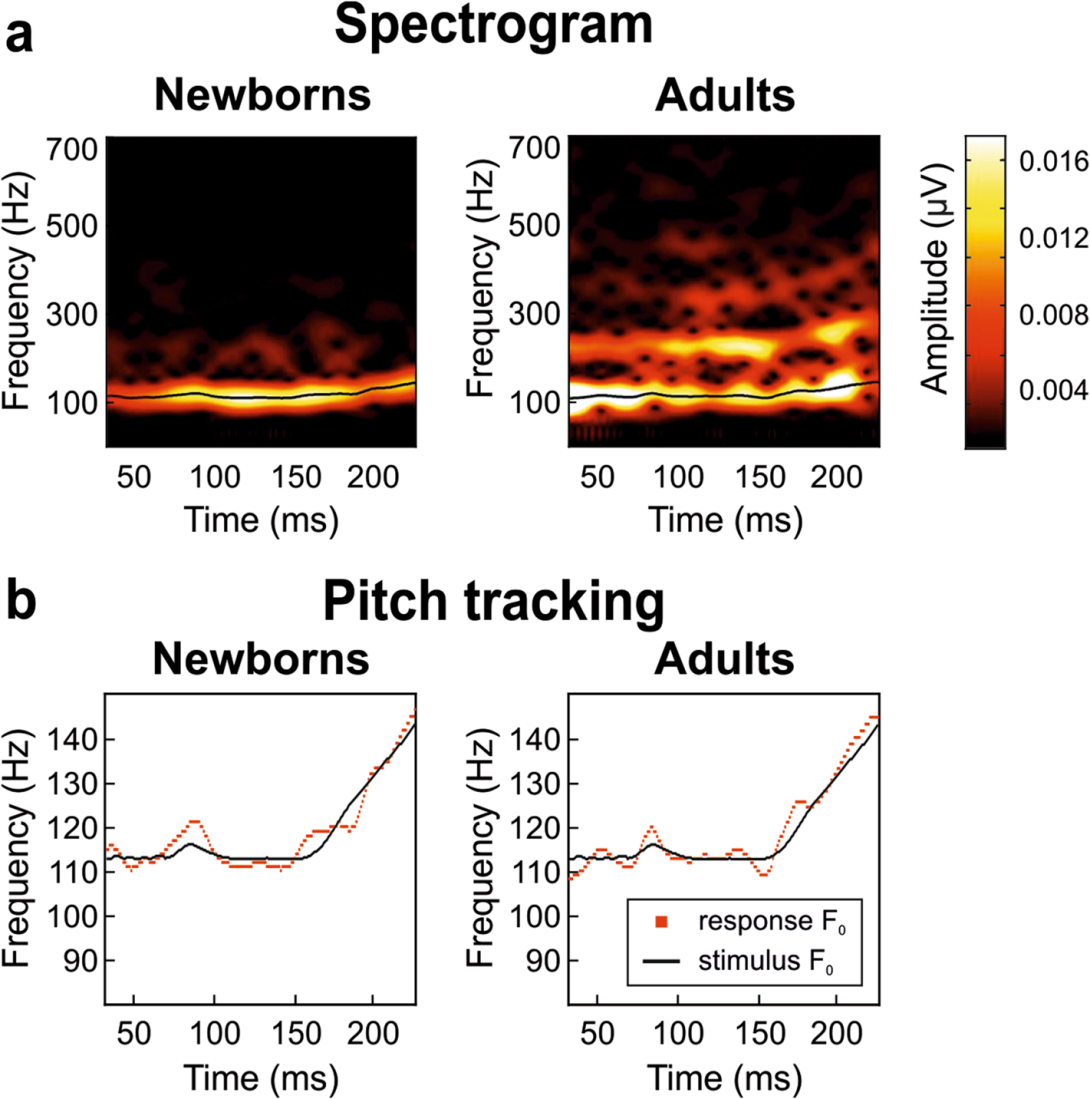
Spectrogram (**a**) and pitch tracking (**b**) extracted from the newborns (left) and adults (right) FFR_ENV_s grand averages elicited by /oa/ stimulus. (**a**) The color scale from white to black represent the spectral amplitude in µV; dark colors represent smallest amplitude values, while light ones represent the highest. (**b**) F_0_s extracted from the stimulus is represented in solid black line, F_0_s extracted from the FFR_ENV_s elicited by the stimulus depicted in dotted red line.

We then computed the *pitch error* per pitch section separately, in order to determine pitch-tracking accuracy of the F_0_ contour^11,29^. Neither significant group or stimulus section differences nor group per stimulus section interaction were found (see Fig. 3a for spectrogram and Fig. 3b for pitch track).

#### Pitch strength

Pitch strength was taken as a measure of periodicity and the magnitude of neural phase-locking of the response^10^ and was also computed separately per stimulus pitch section. Neither significant group or stimulus section differences nor group per stimulus section interaction were found.

### Assessment of formant structure encoding from FFR_TFS_

**Figure 4:**
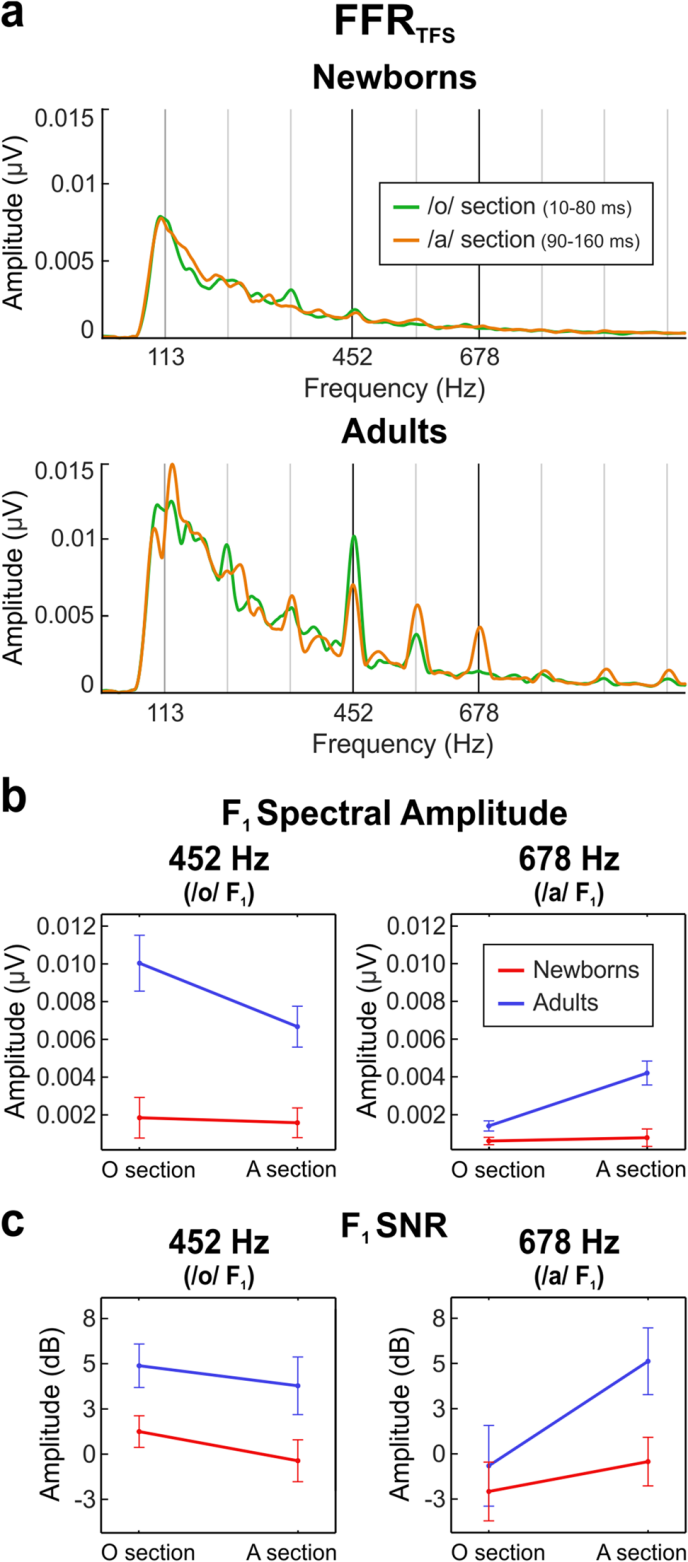
Formant structure encoding in newborns and adults. (**a**) Amplitude FFR_TFS_ spectra extracted from the /o/ vowel section (green) and the /a/ vowel section (orange) from the stimulus, plotted separately for newborns (top) and adults (bottom). (**b**) Main effects graphic of F_1_ spectral amplitude at 452 Hz (left) and 678 Hz (right) during the /o/ vowel section and the /a/ vowel section, plotted in red and blue lines for newborns and adults, respectively. (**c**) Main effects of F_1_ SNR at 452 Hz (left) and 678 Hz (right) during the /o/ vowel section and the /a/ vowel section, depicting neural responses from newborns (red) and adults (blue).

In order to determine the ability of the participants to encode the formant structure of speech sounds, the /oa/ stimulus featured two sections with steady pitch but differing in their formant structure: the /o/ section (10–80 ms; F_1_ = 452 Hz) and the /a/ steady pitch section (90–160 ms; F_1_ = 678 Hz). In order to emphasize temporal fine structure components of the response while diminishing the contribution of responses to the temporal envelope, we computed the FFR_TFS_^29,31^. Grand-average FFR_TFS_ waveforms are shown in Fig. 1c for both groups separately. The frequency spectrum of the /o/ section and the /a/ steady pitch section are shown in Fig. 4a for both groups. All descriptive statistics can be found in Table 2.

**Table 2:** Descriptive statistics for FFR_TFS_ derived parameters: spectral amplitude and SNR for each formant peak frequency (452 Hz; 678 Hz) computed separately during the two vowel sections (/o/; /a/).

Measure	Mean	SD	Median	Q_1_	Q_3_	IQR	Minimum	Maximum
**Spectral amplitude /o/ section at 452 Hz (µV)**								
Newborns	0.0019	0.0010	0.0017	0.0010	0.0024	0.0014	0.0004	0.0053
Adults	0.0100	0.0052	0.0087	0.0065	0.0134	0.0069	0.0030	0.0211
**Spectral amplitude /a/ steady section at 452 Hz (µV)**								
Newborns	0.0016	0.0011	0.0014	0.0006	0.0022	0.0016	0.0002	0.0046
Adults	0.0067	0.0036	0.0068	0.0034	0.0091	0.0057	0.0011	0.0145
**Spectral amplitude /o/ section at 678 Hz (µV)**								
Newborns	0.0006	0.0004	0.0005	0.0004	0.0008	0.0004	<0.0001	0.0017
Adults	0.0014	0.0008	0.0015	0.0008	0.0018	0.0010	0.0003	0.0038
**Spectral amplitude /a/ steady section at 678 Hz (µV)**								
Newborns	0.0008	0.0005	0.0008	0.0004	0.0011	0.0007	0.0001	0.0023
Adults	0.0042	0.0022	0.0038	0.0024	0.0063	0.0039	0.0001	0.0087
**SNR /o/ section at 452 Hz**								
Newborns	1.33	3.22	2.11	− 0.18	4.03	4.21	− 7.10	5.87
Adults	5.20	1.24	5.37	4.58	6.38	1.80	2.18	6.63
**SNR /a/ steady section at 452 Hz**								
Newborns	− 0.39	4.11	0.74	− 3.37	2.75	6.13	− 11.25	5.25
Adults	4.02	2.31	4.81	3.36	5.54	2.17	− 2.21	6.39
**SNR /o/ section at 678 Hz**								
Newborns	− 2.08	4.80	− 1.26	− 4.59	1.40	5.99	− 19.01	3.37
Adults	− 0.66	4.62	0.66	− 2.28	1.93	4.21	− 11.35	5.86
**SNR /a/ steady section at 678 Hz**								
Newborns	− 0.43	4.75	0.90	− 3.84	2.84	6.68	− 15.68	5.38
Adults	5.12	1.14	5.23	4.21	6.05	1.84	3.14	6.95

*SD* standard deviation, *Q*_*1*_ first quartile (25th percentile), *Q*_*3*_ third quartile (75th percentile), *IQR* interquartile range.

Spectral amplitudes and SNRs from the FFR_TFS_ were retrieved separately from neural responses during the /o/ section (10–80 ms) and the /a/ steady pitch section (90–160 ms), selecting the spectral peaks corresponding to stimulus F_1_ frequencies (452 Hz [/o/] and 678 Hz [/a/]), as indicators of the magnitude (absolute and relative) of phase-locking at the selected frequencies.

#### Spectral amplitude at /o/ vowel F_1_

Spectral amplitudes at the /o/ vowel F_1_ (452 Hz) are illustrated in Fig. 4b (left). A main effect of group revealed significantly smaller spectral amplitudes at 452 Hz in newborns as compared to adults (F_(1,50)_ = 85.778, *p* < 0.001, ηp2 = 0.632). A main effect of stimulus section showed a significantly larger spectral amplitude value at 452 Hz during the /o/ vs. /a/ steady pitch sections (F_(1,50)_ = 25.529, *p* < 0.001, ηp2 = 0.338). The group per stimulus section interaction was significant as well (F_(1,50)_ = 18.603, *p* < 0.001, ηp2 = 0.271). *Post-hoc* tests computed to determine the direction of the interaction revealed higher spectral amplitudes in adults at 452 Hz during the /o/ vs. /a/ sections (t_(17)_ = 3.803, *p* = 0.001, Cohen’s *d* = 0.896), but no significant differences were found in newborns.

#### Spectral amplitude at /a/ vowel F_1_

Spectral amplitudes at the /a/ vowel F_1_ (678 Hz) are illustrated in Fig. 4b (right). A main effect of group revealed significantly smaller spectral amplitudes at 678 Hz in newborns as compared to adults (F_(1,50)_ = 79.157, *p* < 0.001, ηp2 = 0.613). A main effect of stimulus section showed a significantly larger spectral amplitude value at 678 Hz during the /a/ steady pitch vs. /o/ sections (F_(1,50)_ = 64.555, *p* < 0.001, ηp2 = 0.564). The group per stimulus section interaction was significant as well (F_(1,50)_ = 50.252, *p* < 0.001, ηp2 = 0.501). *Post-hoc* tests computed to determine the direction of the interaction revealed higher spectral amplitudes in adults at 678 Hz during the /a/ steady pitch vs. /o/ sections (t_(17)_ = − 5.845, *p* < 0.001, Cohen’s *d* = − 1.378), but no significant differences were found in newborns.

#### SNR at /o/ vowel F_1_

SNR values at the /o/ vowel F_1_ (452 Hz) are illustrated in Fig. 4c (left). A main effect of group revealed significantly smaller SNR values at 452 Hz in newborns as compared to adults (F_(1,50)_ = 47.213, *p* < 0.001, ηp2 = 0.486). A main effect of stimulus section showed a significantly larger SNR value at 452 Hz during the /o/ vs. /a/ steady pitch sections (F_(1,50)_ = 4.207, *p* = 0.046, ηp2 = 0.078). No significant group per stimulus section interaction was found.

#### SNR at /a/ vowel F_1_

SNR values at the /a/ vowel F_1_ (678 Hz) are illustrated in Fig. 4c (right). A main effect of group revealed significantly smaller SNR values at 678 Hz in newborns as compared to adults (F_(1,50)_ = 17.136, *p* < 0.001, ηp2 = 0.255). A main effect of stimulus section showed a significantly larger SNR value at 678 Hz during the /a/ steady pitch vs. /o/ sections (F_(1,50)_ = 15.414, *p* < 0.001, ηp2 = 0.236). The group per stimulus section interaction was significant as well (F_(1,50)_ = 4.753, *p* = 0.034, ηp2 = 0.087). *Post-hoc* tests computed to determine the direction of the interaction revealed higher SNR values in adults at 678 Hz during the /a/ steady pitch vs. /o/ sections (t_(17)_ = − 5.656, *p* < 0.001, Cohen’s *d* = − 1.333), but no significant differences were found in newborns.

It should be noted that some SNR values, especially those of newborns at 678 Hz peak, were very close to zero. In order to ascertain whether there was a measurable signal when expected (at 452 Hz during the /o/ section and at 678 Hz during the /a/ section), we submitted the SNR values, per group and per condition separately, to one-tailed, one sample t-tests against zero. Results demonstrated that newborns had a measurable signal for lower frequency formants, as shown by significant differences in SNR at 452 Hz during the /o/ section (t_(33)_ = 2.407, *p* = 0.022, Cohen’s *d* = 0.414), but no clear response at 678 Hz during the /a/ section (*p* = 0.602). Intriguingly, the SNR value at 678 Hz during the /o/ section was negative and significantly different from zero (mean ± SD: − 2.08 ± 4.80 dB; t_(33)_ = − 2.530, *p* = 0.016, Cohen’s *d* = − 0.433). Adult participants exhibited a measurable signal in the two conditions: at the 452 Hz peak during the /o/ section (t_(17)_ = 17.737, *p* < 0.001, Cohen’s *d* = 4.194) and at the 678 Hz during the /a/ section (t_(17)_ = 19.043, *p* < 0.001, Cohen’s *d* = 4.491).

## Discussion

We hereby provide an in-depth characterization of the neural encoding of speech sound features that newborns exhibit during their first hours of life, by comparing FFRs from healthy newborns and normal-hearing adult participants elicited by a novel, two-vowel /oa/ stimulus, with a rising pitch ending. Regarding the FFR parameters indexing voice pitch encoding, extracted from the FFR_ENV_, our results support previous findings showing no significant differences in voice pitch encoding ability at birth as compared to adults, as can be appreciated from the SNR values at F_0_ peak as well as in pitch tracking measures, such as stimulus-to-response cross-correlation, pitch error and pitch strength. Concerning the FFR parameters indexing formant structure encoding, extracted from the FFR_TFS_, as expected, newborns exhibited overall diminished amplitudes than adults at both F_1_ peaks of interest (452 and 678 Hz). On the other hand, obtained SNR values in newborns were higher at 452 Hz (/o/ F_1_) during the /o/ section than during the /a/ section but not different at 678 Hz (/a/ F_1_), revealing the functional state of formant structure encoding mechanisms, which appear to be partially developed but still to mature, especially at higher frequency ranges. Furthermore, our results prove the feasibility to record and assess simultaneously both voice pitch and formant structure encoding within a thirty-minute period, a time-span compatible with clinical settings that allows obtaining the FFR_ENV_ and the FFR_TFS_ in large samples of newborns.

### Considerations on the mother’s womb acting as an acoustic filter and speech perceptual skills at birth

Speech perception abilities are crucial for early phonetic discrimination^1,5,6,58^. Human hearing begins approximately at the 26th week of fetal life and most of the development takes place between the 26th and 28th week of gestation^54,59–62^, when hair cells and their connections to the cochlea are mature enough to tune in to specific frequencies. In this regard, previous research showed that fetuses can hear and remember language sounds and may learn about several sound properties while in the womb^61^. Studies in newborns have shown a preference for their mother’s voice^63^ and for their native language^64,65^, as well as behavioral recognition of children’s stories heard only during pregnancy^66^. But, what speech sound features do babies rely upon to exhibit such identification skills? Considering that the mother’s womb acts as a low-pass filter, the sounds available to a fetus during the gestation period are dominated by a low frequency content (< 500 Hz^5,67–69^), while higher frequency ranges, which characterize most of the temporal fine structure of speech^46,70^, would only be fully available at birth. Indeed, neonates may base their preferences on pitch contours and slow temporal dynamics, features available during pregnancy^71–73^. Furthermore, albeit previous studies have shown neural signatures of vowel change detection for vowel pairs differing only in second formant (F_2_) frequencies in newborns^20^ and 6 months-old babies^74^, recent electrophysiological^5^ and behavioral^75^ evidence suggests that infant vowel discrimination relies more strongly on F_1_ (usually below 800 Hz) than F_2_ frequency differences. For instance, in a comprehensive study, McCarthy et al.^5^ analyzed neural responses to vowel changes using all pairs of a set of 7 English vowels, and showed that phonetic development from 4 to 11 months-old exhibits an increasing sensitivity to higher-frequency acoustic information (i.e., infants progressively rely less on F_1_ changes and more on F_2_ changes). Importantly, while youngest infants (4–5 months-old) neural responses appeared to reflect vowel acoustics (i.e., larger acoustic changes were reflected by larger neural response changes), those from older infants (10–11 months-old) seemed to represent putatively categorical changes (i.e., vowel space maps recreated from neural data showed large differences between vowel pairs with small acoustic differences). Intriguingly, a close inspection of their data (particularly at Fig. 4) strongly suggests that vowel pairs with lower F_1_ frequency content (/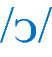 / vs. /u/; < 500 Hz) are represented in youngest infants’ vowel space farther apart from each other than vowel pairs with higher F_1_ frequency content (/a/ vs. /ε/; > 500 Hz), a pattern not apparent in older infants. However, the authors did not explicitly test this hypothesis. In fact, to the best of our knowledge, there is no behavioral or neurophysiological study in newborns or young infants explicitly testing vowel discrimination as a function of formant frequency. This may constitute an exciting avenue for future research linking auditory neural responses to auditory pathway and vowel discrimination development.

Regarding our data, in view of the above and taking into account that 1) the chosen first formants of our stimulus fall below (/o/ F_1_) and above (/a/ F_1_) the 500 Hz filter cut-off; 2) FFR spectral amplitudes increase with age^57^; 3) FFR spectral amplitudes diminish along the frequency axis^55^; and 4) FFRs are plastically modulated by experience^9,51,57,68^, it appears reasonable to expect certain degree of response in newborns at the lower frequency formant (452 Hz) and a fast decay of spectral power at the higher frequency formant (678 Hz). In any case, it seems plausible that certain speech sound processing skills were already mature at birth due to a greater exposure during pregnancy, while others would still be undeveloped.

### Functional maturity state differences across speech perceptual skills at birth

A first indicator of auditory system’s functional maturity is auditory transmission delay^76,77^. Measuring wave V latencies and stimulus-to-response neural lags (which were consistent with activity generated in the brainstem^76^) we found, in agreement with previous literature, shortened delays in adult participants, which may be due to the increasing myelination and age-related changes in synaptic function^13,42,43,53^.

However, even with a still maturing transmission speed, our results demonstrate that newborns accurately encode the F_0_ of speech sounds as well as track changes in voice pitch during immediate postnatal hours, in line with previous studies^8–13^. Although spectral amplitudes at the F_0_ peak were smaller in newborns as compared to adults, no significant differences were found with the adult sample when choosing relative amplitude measurements (i.e., SNR). Thus, the higher spectral amplitude values for adults could be due to the fact that, even during the pre-stimulus period, they also presented a higher spectral noise level (pre-stimulus root mean square: newborns = 0.03 ± 0.01 µV; adults = 0.05 ± 0.02 µV; U_(50)_ = 571, *p* < 0.001).

On the other hand, our results indicate a differential processing of formant structure in newborns in comparison to adults. Similar to the results on the FFR_ENV_, neonates showed significantly smaller FFR_TFS_ absolute spectral amplitude values, but also smaller relative measures such as the SNR. However, our data demonstrate that newborns can encode the fine structure of speech sounds to a certain extent, with some limitation for higher frequency ranges, as evidenced by the fact that their SNR values were higher at 452 Hz (/o/ F_1_) during the /o/ section than during the /a/ section, but at 678 Hz (/a/ F_1_) they were not significantly different from zero. Although the SNR at 678 Hz during the /o/ section was negative in newborns, when analyzing the amplitude of the frequency spectrum (Fig. 4a) we observed that spectral amplitudes at 678 Hz during either of the two sections were very weak. Because of the reduced spectral amplitude and its large standard deviation, we considered this negative value as negligible, probably due to a noisy signal at higher frequencies rather than to active inhibition.

We considered the possibility that our results regarding formant structure encoding could be influenced by the internal structure of the stimulus, i.e., the /o/ section always preceded the /a/ section. As infants and neonates seem to preferentially use rhythmic cues to segment syllables and words from the acoustic stream^78,79^, newborns may be more sensitive to sound onsets than codas. According to the temporal sampling framework hypotheses, put forward by Goswami^80^, rhythmic amplitude envelope modulations would entrain cortical oscillatory activity to exert a preferential processing of syllable onsets. However, there is no obvious reason why such preferential onset processing should be apparent only at formant structure encoding and not at pitch encoding. Therefore, in order to shed some light on this possible confounding factor, we decided to statistically compare the SNR values at F_0_ during the /o/ steady pitch section (10–80 ms) vs. the /a/ steady pitch section (90–160 ms), using a paired-samples t-test for each group of age. Our results showed that there were no significant differences in the SNR values at F_0_ between stimulus steady pitch sections for either of the two groups (newborns: t_(33)_ = − 1.466, *p* = 0.152, Cohen’s *d* = -0.251; adults: t_(17)_ = 0.797, *p* = 0.436, Cohen’s *d* = 0.188; for further statistical information, the reader is referred to Suppl. Table 1). Thus, no onset effect in pitch encoding was observable in any group. Moreover, given the rhythmic stimulation used in our study (SOA = 295 ms), half cycle of an entrained oscillation would last enough to cover, with the high excitability phase, both /o/ and /a/ steady pitch sections of our stimulus. Furthermore, the high frequency ranges we are dealing with in our FFR data (beyond 100 Hz) are more prone to elicit recordable subcortical activity than cortical^81–83^, and the modulation of phase-locking in subcortical neuronal ensembles by cortical oscillations has not been described, to the best of our knowledge, in the literature. Finally, in our study, the adult FFR_TFS_ SNR values at the formant peaks showed a double dissociation, being larger at the /o/ F_1_ frequency during the /o/ section and at the /a/ F_1_ frequency during the /a/ section, ruling out any onset effect. Therefore, given the pattern of results and the reviewed literature, an onset effect seems a negligible influencing factor in our results. In any case, further research studying the influence of vowel order should be carried out to help better clarify this possible confound (e.g., presenting an /ao/ syllable and comparing the pattern of results).

These results thus agree with the abovementioned notion that, due to the low-pass filter characteristics of the womb, fetuses are probably isolated from the mid and high frequency acoustic content of external sounds that characterizes most of the temporal fine structure of speech^46,70^. Yet, while lacking the required prior experience for a mature perceptual system responding accurately to high frequencies, the ability to encode fine structure per se seems to be present at birth. Future testing with premature babies early exposed to natural sounds may shed more light on this issue.

Overall, our results are in line with the idea that humans, despite their limited experience to speech at birth, present mature functional mechanisms to detect changes in speech features at an unexpectedly early age^8,84^, and since alterations in the neural mechanisms underlying temporal envelope encoding are associated to several disabilities such as autism^48^, dyslexia^70^ or other learning problems^33^, it is tempting to speculate that the encoding of temporal envelope information, such as its periodicity, may play a crucial role in the very first stages of language acquisition^8^. Temporal envelopes could provide a neural synchrony channel onto which separate neural representations of other speech features would anchor as parts of an ensemble that would, ultimately, give rise to a coherent unitary entity^85^. Furthermore, there is increasing evidence that the FFR is a brain response that receives subcortical and cortical contributions in a frequency-specific manner, with frequencies below 150 Hz originating mainly from subcortical sources^30,82,83,86,87^. Therefore, it is tempting to speculate that the effects observed here may reflect the increasing maturation of the subcortical auditory system from birth to adulthood.

The reported differences in formant structure encoding abilities found between newborns and adults open a window of opportunity to study the developmental progression of these skills. Considering that the gradual increase of phase-locking to high-frequencies is age-dependent^42^, understanding how inter-individual differences in development as revealed by FFR_TFS_ neural responses relate to the acquisition of formant encoding perceptual skills could be used to identify potential risks of future disabilities. Early impairment detection is thus critical to allow early interventions and to maximize the development of speech and listening competences, essential requirements for the acquisition of optimal literacy skills^15^.

### Considerations on speech stimuli commonly used for newborn FFR studies

In language FFR studies, the most commonly applied speech stimuli are mandarin syllables following the four different lexical tones^8,13,48,51,52,88,89^, and different single vowels with rising pitch^9,10,43,90^. The use of these stimuli focused the research field on assessing voice pitch encoding, putting the assessment of formant structure encoding aside. A notable exception is the widely used consonant–vowel syllable /da/^11,14,42,44,45,49,56,91,92^, which contains a fine structure change during the consonant–vowel transition. The relevance of using this stimulus relies on the fact that stop consonants are an important constraint in populations with literacy impairments^93^, and since stop bursts are rapid and low in amplitude in the /d/ consonant compared to vowels, even normal-hearing adults and children can find difficult to discriminate it from other contrastive stop consonants^28^. However, the short duration of the consonant transition and the high (and changing) frequency peak of the formants that compose it (e.g., the difference between /d/ and /g/ appears in the second formant: /da/ F_2_ = 1438–1214 Hz, /ga/ F_2_ = 1801–1214 Hz), render this type of stimuli suboptimal in the characterization of FFR responses, which exhibit a spectral power decay with increasing frequency^55^, especially in populations with an immature encoding of the high frequency content of sounds, such as newborns^39,42^. Hence, while the phase locking to lower frequency sounds could in principle be safely assessed from the first hours of life^42,50^ as we demonstrate here as well, the lack of prenatal experience to the high frequency content of sounds and the requirement of a later and greater maturation of the auditory system to encode them^39,42,46,50^ pose some limitations in the design of stimuli suited to study formant structure encoding.

Therefore, we believe our newly designed /oa/ stimulus, with pitch variation and two vowel sections with different formant structure based on relatively lower frequency harmonic components and suitable durations for accurate spectral analyses, enables a proper assessment of speech sound temporal envelope (FFR_ENV_) and temporal fine structure (FFR_TFS_) encoding.

## Conclusion

The present study provides the first evidence that neonates are able to encode not only the voice pitch of speech sounds and its changes with great accuracy, as has been demonstrated in previous research, but also the formant structure. Specifically, newborns show emerging formant structure encoding skills at lower frequency ranges but still immature encoding precision at higher frequency ranges. In addition, having already proved the feasibility of successfully recording temporal envelope and temporal fine structure in newborns, we here promote the use of this new stimulus as a powerful tool to perform a longitudinal assessment of speech encoding in babies from their very first hours of life throughout the first years of infant development.

## Methods

### Participants

A sample of 34 healthy term newborns (17 females; mean gestational age = 40.19 ± 1.08 weeks; mean birth weight = 3379 ± 289 g; aged 14–78 h after birth) was recruited from Sant Joan de Déu Hospital in Barcelona (Spain). Obstetric pathologies, high-risk gestations and risk factors related to hearing impairments (according to the criteria of the Joint Committee of Infant Hearing^94^) were considered excluding factors. All newborns had Apgar scores higher than 8 at 1 and 5 min of life and had passed the standardized hearing screening test based on the automated auditory brainstem response system (ALGO 3i, Natus Medical Incorporated, San Carlos, CA). Six additional newborns were attempted to be recorded but finally not included in the study because they woke up before concluding the recording session, and it was not possible to help them falling asleep again.

Additionally, 18 healthy young adult participants (14 females; mean age = 26.94 ± 3.78 years) with no self-reported history of neurological, psychiatric or hearing impairment, and with normal or corrected-to-normal visual acuity were included in the study for comparison. Taking into account previous research showing no differences between sexes for the encoding of frequencies until 720 Hz^95,96^, chances that data extracted from our selected range of analyzed frequencies (up to 678 Hz) were affected by sex condition were low. All participants underwent a screening pure tone audiometry to ensure a normal hearing level at 250, 500, 1000, 2000 and 4000 Hz. Excluding factors were mean hearing thresholds above 25 dB sound pressure level (SPL) or mean interaural hearing threshold differences larger than 20 dB SPL.

Both newborns and adults underwent a standard click-evoked auditory brainstem response test employing a standard *SmartEP* platform (Intelligent Hearing Systems, Miami, Fl, USA), with a 100 µs square-wave click stimulus delivered at 65 dB SPL for adults and 60 dB SPL for newborns. Following the precedent of Jeng et al.^97^, differences in stimulus intensities were chosen to compensate for the smaller ear canal volumes observed in young infants^98,99^. All participants included in the sample had a reliably identifiable wave V. The mean latency of wave V was 8.70 (± 0.42 SD) ms for newborns and 6.54 (± 0.39 SD) ms for adults, and its mean amplitudes were 0.13 (± 0.08 SD) µV for newborns and 0.29 (± 0.12 SD) µV for adults (Suppl. Fig. 1). All these values were comparable to those published previously^11,100^.

The study was approved by the Ethical Committee of Clinical Research (CEIC) of the Sant Joan de Déu Foundation (Approval ID: PIC-53-17) and the Bioethics Committee of the University of Barcelona, and all adult participants and newborns’ legal guardians gave informed consent in compliance with the Code of Ethics of the World Medical Association (Declaration of Helsinki). The data that support the findings of this study and the code used for data analysis are available upon reasonable request to the authors.

### Stimulus

Inspired by the aforementioned previous stimuli limitations (e.g., short duration of consonant transitions and changing formants, high frequency content), a 250 ms two-vowel syllable stimulus with a rising pitch ending (/oa/) was created in *Praat*^101^ (Fig. 1a). The /o/ vowel section (F_1_ = 452 Hz; F_2_ = 791 Hz) lasted from 0 to 80 ms, the /a/ vowel section (F_1_ = 678 Hz; F_2_ = 1017 Hz) from 90 to 250 ms, and the /oa/ formant transition section from 80 to 90 ms. Stimulus pitch was kept steady at F_0_ = 113 Hz from 0 to 160 ms and increased linearly up to 154 Hz from 160 to 250 ms. We used 113 Hz F_0_ instead of the common 100 Hz F_0_ to avoid electric line noise harmonics by the European 50 Hz alternating current^11^. In order to maximize the detection of differences in vowel formant encoding in the FFR_TFS_, formant peak frequencies coincided with harmonics of the fundamental.

Stimuli were delivered monaurally to the right ear with a stimulus-onset asynchrony (SOA) of 295 ms, in alternating polarities, at an intensity of 65 dB SPL for adults (Etymotic shielded earphones of 300 Ω, ER, Elk Grove Village, IL, USA) and 60 dB SPL for newborns (same earphones connected to a Flexicoupler disposable adaptor, Natus Medical Incorporated, San Carlos, CA) using Intelligent Hearing Systems (Miami, Fl, USA). Differences in stimulus intensities were chosen for the same reason as in click stimulus.

### Procedure

All newborns were recorded at the hospital room where they were resting with their mother. After the neonate passed the universal hearing screening test, the researcher started the recording session as soon as the newborn fell asleep, interrupting it to any sign of discomfort or sleep disruption and resuming it when the newborn was asleep again. The total mean duration of a test session was approximately 25 min (two click blocks × 2000 sweeps × 51.81 ms SOA, plus four /oa/ stimulus blocks × 1000 sweeps × 295 ms SOA, plus the duration of rejected sweeps), plus recording preparation time (around 5 min). Adult participants were tested in an acoustically shielded chamber in a laboratory facility located at the University of Barcelona, following the same procedure as in newborns with the exception of being awake with their eyes closed. Taking into account that the analyzed frequency content of neural responses recorded in the present study belongs to a higher frequency range than those characteristic of cortical sources (beyond 100 Hz^81^), and that attentional modulations of the FFR seemingly affect only cortical sources^30,86,102,103^, we can consider the contribution of alertness as a confounding factor in our results to be rather weak.

### Data acquisition

FFRs were recorded from both newborns and adults with a *SmartEP* platform including the *cABR* and *Advanced Hearing Research* modules connected to a *Duet* amplifier (Intelligent Hearing Systems, Miami, Fl, USA), using three disposable snap Ag/AgCl electrodes placed in a vertical montage (ground electrode at the forehead; active at Fpz; online reference at the right mastoid, ipsilateral to the stimulated ear). All electrode impedances were kept < 7 kΩ. The continuous signal was acquired at a sampling rate of 13,333 Hz with an online bandpass filter from 30 to 1500 Hz and epoched from − 40.95 (pre-stimulus period) to 249.975 ms relative to stimulus onset. A total of 4000 artifact-free responses were obtained for each participant after automatic rejection of any sweep with voltage values exceeding ± 30 µV.

### FFR processing

Data was bandpass filtered offline from 80 to 1500 Hz. In order to assess voice pitch encoding, it was necessary to accentuate the FFR components corresponding to the encoding of the stimulus envelope, such as the fundamental frequency (F_0_). Thus, neural responses were averaged by adding sweeps corresponding to the two stimulus polarities [(Rarefaction + Condensation)/2], yielding the envelope-following response (FFR_ENV_). This procedure also aids in minimizing the contribution of putative cochlear microphonics. On the other hand, to properly evaluate formant structure representation, it was necessary to emphasize the FFR components highlighting the encoding of the stimulus temporal fine structure, such as vowel formants (F_1_, F_2_), and minimize the contribution of activity related to the envelope. To this aim, the responses to stimuli of alternating polarities were subtracted [(Rarefaction–Condensation)/2], yielding the temporal fine structure-following response (FFR_TFS_)^29,31^. In this study, only the FFR_TFS_ spectral peaks corresponding to F_1_ frequencies were analyzed, since those from F_2_ frequencies belonged to a very high frequency range that elicits weak neural responses difficult to record and, therefore, could not be reliably observed in all participants, especially in newborns. All data were analyzed using MATLAB R2019b^104^.

### FFR parameters and statistical analysis

To give a comprehensive description of FFR properties both in newborns and adults, we computed several parameters, which we briefly detail below (see Ribas-Prats et al.^11^ for a full description of procedure, scripts and routines). All statistical analyses were performed on SPSS 25.0^105^. Descriptive statistics are shown as mean, standard deviation (SD), median, first (Q_1_) and third (Q_3_) quartiles, interquartile range (IQR), and minimum and maximum values of the parameters for each group of age. The Kolmogorov–Smirnov test with the Lilliefors’ significance correction was selected to check the normal distribution of the samples. Results were considered significant when *p* < 0.05. Contrast statistics, as well as *p* values and effect sizes obtained from statistically significant comparisons are reported in the Results sections. Statistically non-significant results and normality tests are reported in Suppl. Table 1.

### Neural transmission delay

#### Neural lag

Neural lag was taken as an estimation of FFR latency due to the auditory system’s neural transmission delay^11^, and was extracted from a cross-correlation of the entire stimulus with the neural response (10–250 ms), selecting the time lag that corresponds to the maximum cross-correlation value. The obtained values were non-normally distributed, so a Mann–Whitney U test was used to assess for significant group differences (i.e., whether newborns showed a different transmission delay than adults).

### Voice pitch encoding

To determine the abilities of newborns (by comparison with adults) to encode the voice pitch contour of the auditory stimulus presented, several parameters were extracted from the FFR_ENV_:

#### Spectral amplitude at F_0_ peak

Spectral amplitude at F_0_ peak (113 Hz) was calculated as an indicator of the magnitude of neural phase-locking at that specific frequency^49^ only during the steady pitch section of the stimulus (10–160 ms), due to the continuous variation in pitch frequency throughout the rising section (160–250 ms). Since the obtained values were normally distributed, we employed a two-samples T-test to assess for significant group differences (i.e., whether newborns showed different spectral amplitudes of the signal at F_0_ peak than adults).

#### Signal-to-noise ratio

Signal-to-noise ratio (SNR) at F_0_ peak was taken as an estimation of the relative spectral magnitude of the response, taking into account not only the amplitude value of the signal at the frequency peak of interest (113 Hz) but also around that peak. Therefore, we divided the mean amplitude within a ± 5 Hz frequency window centered at the peak of the frequency of interest (F_0_) by the mean amplitude within two 28 Hz wide frequency windows (flanks) centered at ± 19 Hz from the frequency of interest (e.g., for F_0_ = 113 Hz, the mean amplitude from 108 to 118 Hz divided by the average of the mean amplitude from 80 to 108 Hz and the mean amplitude from 118 to 146 Hz). In order to ascertain group differences in the magnitude of the F_0_ encoding and discern whether newborns had different responses to voice pitch than adults, we used Mann–Whitney U tests because the obtained values were non-normally distributed.

#### Stimulus-to-response cross-correlation

In order to assess the accuracy with which the FFR_ENV_ reproduces the stimulus waveform, we calculated the normalized cross-correlation between each individual’s neural response and the stimulus, separately for the /a/ steady (90–160 ms) and /a/ rising pitch contour stimulus sections (160–250 ms)^29^. The maximum value reached within a time lag of 3 to 10 ms (corresponding to the neural lag) was selected (Pearson’s r; values from − 1 to 1). The obtained values were non-normally distributed. Therefore, to test for putative between-subjects differences (i.e., whether newborns showed a different overall stimulus–response correlation than adults), a Mann–Whitney U test was used, with Age (newborns; adults) as grouping variable and Stimulus Section (/a/ steady; /a/ rising) as contrast variable. To test for putative within-subjects differences (i.e., whether stimulus–response correlations were different depending on stimulus pitch contour), a Wilcoxon test for two related samples comparing the correlation values obtained for each stimulus section (/a/ steady; /a/ rising) was used. Finally, to test for a putative interaction between factors (i.e., whether newborns showed a different correlation value depending on stimulus pitch section than adults), a Mann–Whitney U test was used taking Age (newborns; adults) as grouping variable and the difference between the two conditions of the Stimulus Section (/a/ steady – /a/ rising) as contrast variable.

We also computed the normalized autocorrelation of the neural response, as well as that of the stimulus, in 40 ms sliding bins, to extract pitch error and pitch strength values.

#### Pitch error

Pitch error per stimulus section was used to determine pitch-tracking accuracy of the F_0_ contour^11,29^ (corresponding to the autocorrelation peak lag per bin) by averaging the absolute Euclidian distance between the stimulus F_0_ contour and the response F_0_ per pitch section separately (steady [10–160 ms]; rising [160–250 ms]; starting from the onset of the section + the individual neural lag; values in Hz). Since obtained values were non-normally distributed, to determine between-subject effects, within-subjects effects and interaction, we followed the same procedure as with the stimulus-to-response cross-correlation explained above.

#### Pitch strength

Pitch strength per stimulus section was taken as a measure of periodicity and the magnitude of neural phase-locking of the response^10^, and calculated by averaging the obtained peak autocorrelation value of the response across bins, per pitch section separately (steady; rising; starting from the onset of the section + the individual neural lag; values from − 1 to 1). Values were non-normally distributed, thus an identical method with the same factors as employed above in cross-correlation and pitch error parameters was used to determine between-subject effects, within-subjects effects and interaction.

### Formant structure encoding

Regarding the encoding of the perceptual quality of formant structure, several parameters were retrieved from the FFR_TFS_.

#### Spectral amplitude

Spectral amplitudes at spectral peaks corresponding to stimulus F_1_ frequencies (452 Hz [/o/] and 678 Hz [/a/]) were retrieved separately from neural responses to the /o/ section (10–80 ms) and the /a/ steady section (90–160 ms). All values were normally distributed, so an ANOVA test was conducted. Regarding the spectral amplitude at 452 Hz, (a) the Group variable (newborns; adults) was chosen as between-subjects factor, to examine whether newborns showed different amplitude values at 452 Hz than adults; (b) Stimulus Section (/o/ section; /a/ section) as within-subjects factor, in order to test whether spectral amplitudes at 452 Hz were different depending on stimulus vowel section; (c) Interaction between factors was analyzed to ascertain whether newborns showed a different amplitude value at 452 Hz depending on stimulus vowel section than adults. Pursuing an identical purpose, we conducted again the same test to examine differences at 678 Hz. The transition from /o/ vowel to /a/ vowel was not analyzed due to its short duration (10 ms).

#### Signal-to-noise ratio

Following the same procedure as with the spectral amplitude, SNRs at spectral peaks corresponding to stimulus F_1_ frequencies (452 Hz [/o/] and 678 Hz [/a/]) were also retrieved separately from responses to the /o/ and the /a/ steady section, using an identical method to calculate it as described above for the FFR_ENV_. All values were normally distributed, so ANOVA tests on 452 Hz and 678 Hz were conducted with the same factors and objectives as described above for F_1_ spectral amplitudes analyses.

All analyses were additionally computed by excluding participants with extreme values (more than three interquartile ranges; N = 9; 4 newborns + 5 adults). As the statistical results obtained did not alter the main findings of the study, we decided to keep all participants within the reported analyses to better represent the inherent variability of our samples (results excluding extreme values are reported in Suppl. Tables 2–7).

## Supplementary Information

Supplementary Table S1.

Supplementary Information.
