# Supplementary material for: Neural encoding of voice pitch and formant structure at birth as revealed by frequency-following responses
Source: Sci Rep. 2021 Mar 23;11:6660. doi: 10.1038/s41598-021-85799-x (PMC7987955; doi:10.1038/s41598-021-85799-x)
Supplement: Supplementary file 2 — Supplementary Information. [file 41598_2021_85799_MOESM2_ESM.docx]

**Neural encoding of voice pitch and formant structure at birth as revealed by frequency-following responses**

Sonia Arenillas-Alcón^1,2,3^, Jordi Costa-Faidella^1,2,3,^*, Teresa Ribas-Prats^1,2,3^, María Dolores Gómez-Roig^3,4^ & Carles Escera^1,2,3,^*

^1^ Brainlab – Cognitive Neuroscience Research Group. Department of Clinical Psychology and Psychobiology, University of Barcelona (Catalonia, Spain)

^2^ Institute of Neurosciences, University of Barcelona (Catalonia, Spain)

^3^ Institut de Recerca Sant Joan de Déu, Esplugues de Llobregat (Catalonia, Spain)

^4^ BCNatal – Barcelona Center for Maternal Fetal and Neonatal Medicine (Hospital Sant Joan de Déu and Hospital Clínic), University of Barcelona (Catalonia, Spain)

**FIGURES**

**Supplementary Figure 1**


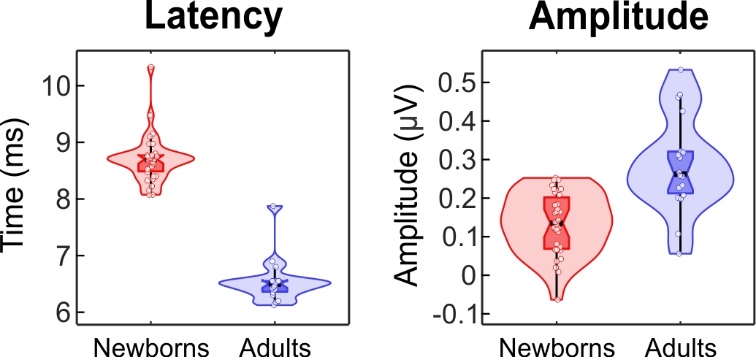


**Figure Legend**

**Suppl. Fig. 1.** Data distribution of wave V parameters for both groups. Scatter plots show all tested participants in each group with violin plots for latency (left) and amplitude (right). In each plot, horizontal black line and vertical black line indicate the median and the interquartile range, respectively.

**TABLES**

**Supplementary Table 1**

Attached Excel file.

**Table Legend**

**Suppl. Table 1.** Detailed statistical analyses on FFR parameters extracted from the FFR_ENV_ and FFR_TFS_, specifying significant and non-significant results and normality tests.

**Supplementary Table 2**

**Suppl. Table 2. Analysis by excluding extreme cases.** Testing for normality distribution of the data by Kolmogorov-Smirnov test.

| Measure, N = 43 | Contrast Statistic | *p* |
| --- | --- | --- |
| **Neural lag** |  |  |
| Newborns | 0.171 | 0.025 |
| Adults | 0.302 | 0.002 |
| **Spectral amplitude F0** (steady part; 10-160 ms) |  |  |
| Newborns | 0.123 | 0.200 |
| Adults | 0.211 | 0.119 |
| **SNR F0** (steady part; 10-160 ms) |  |  |
| Newborns | 0.185 | 0.010 |
| Adults | 0.160 | 0.200 |
| **Cross-correlation (Pearson’s r)** |  |  |
| **/a/ steady section** |  |  |
| Newborns | 0.147 | 0.095 |
| Adults | 0.131 | 0.200 |
| **/a/ rising section** |  |  |
| Newborns | 0.135 | 0.173 |
| Adults | 0.179 | 0.046 |
| **Pitch error (Hz)** |  |  |
| **/a/ steady section** |  |  |
| Newborns | 0.149 | 0.049 |
| Adults | 0.127 | 0.200 |
| **/a/ rising section** |  |  |
| Newborns | 0.152 | 0.074 |
| Adults | 0.165 | 0.200 |
| **Pitch strength (r)** |  |  |
| **/a/ steady section** |  |  |
| Newborns | 0.115 | 0.040 |
| Adults | 0.162 | 0.200 |
| **/a/ rising section** |  |  |
| Newborns | 0.119 | 0.200 |
| Adults | 0.166 | 0.200 |

Data extracted from the FFR_ENV_.

**Supplementary Table 3**

**Suppl. Table 3. Analysis by excluding extreme cases.** Testing for normality distribution of the data by Kolmogorov-Smirnov test.

| Measure, N = 43 | Contrast Statistic | *p* |
| --- | --- | --- |
| **Spectral Amplitude 452Hz_Osection** |  |  |
| Newborns | 0.133 | 0.185 |
| Adults | 0.174 | 0.200 |
| **Spectral Amplitude 452Hz_Asection** |  |  |
| Newborns | 0.124 | 0.200 |
| Adults | 0.174 | 0.200 |
| **Spectral Amplitude 678Hz_Osection** |  |  |
| Newborns | 0.130 | 0.200 |
| Adults | 0.211 | 0.115 |
| **Spectral Amplitude 678Hz_Asection** |  |  |
| Newborns | 0.126 | 0.200 |
| Adults | 0.133 | 0.200 |
| **SNR 452Hz_Osection** |  |  |
| Newborns | 0.145 | 0.110 |
| Adults | 0.149 | 0.200 |
| **SNR 452Hz_Asection** |  |  |
| Newborns | 0.161 | 0.056 |
| Adults | 0.259 | 0.077 |
| **SNR 678Hz_Osection** |  |  |
| Newborns | 0.180 | 0.114 |
| Adults | 0.182 | 0.200 |
| **SNR 678Hz_Asection** |  |  |
| Newborns | 0.200 | 0.200 |
| Adults | 0.134 | 0.200 |

Data extracted from the FFR_TFS_

**Supplementary Table 4**

**Suppl. Table 4. Analysis by excluding extreme cases.** Descriptive statistics and group comparison (newborns vs. adults) for spectral amplitude and SNR for F_0_ = 113 Hz frequency peak during the steady section of the stimulus, extracted from FFR_ENV_.

| Measure | Newborns  (n = 30) | Adults  (n = 13) | T | *p* |
| --- | --- | --- | --- | --- |
| F_0_ Spectral Amplitude | 13.29 (1.26) | 17.57 (2.16) | -1.796 | 0.080 |
| F_0_ SNR | 4.99 (0.84) | 4.38 (0.93) | 158^a^ | 0.339 |

Results are expressed as mean (SD). ^a^ Mann-Whitney U test.

**Supplementary Table 5**

**Suppl. Table 5. Analysis by excluding extreme cases.** Main effects and interactions analyses from FFR_ENV_ derived parameters.

| Measure, N = 43 | Contrast Statistic | *p* |
| --- | --- | --- |
| **Cross-correlation (Pearson’s r)** |  |  |
| Main effect GROUP | 0.022 | 0.882 |
| Main effect STIMSECTION | 66.810 | < 0.001 |
| Interaction | 0.558 | 0.459 |
| **Pitch error (Hz)** |  |  |
| Main effect GROUP | 0.331 | 0.568 |
| Main effect STIMSECTION | 1.138 | 0.292 |
| Interaction | 0.023 | 0.880 |
| **Pitch strength (r)** |  |  |
| Main effect GROUP | 1.921 | 0.173 |
| Main effect STIMSECTION | 0.589 | 0.447 |
| Interaction | 0.781 | 0.382 |

Group = Newborns; Adults. StimSection = /a/ steady; /a/ rising.

**Supplementary Table 6**

**Suppl. Table 6. Analysis by excluding extreme cases.** Descriptive statistics and group comparison (newborns vs. adults) for spectral amplitude and SNR for each formant frequency peak (452 Hz; 678 Hz) during the /o/ and the /a/ steady sections, extracted from FFR_TFS_.

| Measure | Newborns  (n = 30) | Adults  (n = 13) | T | *p* |
| --- | --- | --- | --- | --- |
| Spectral Amplitude 452Hz_Osection | 1.958 (0.195) | 9.946 (1.633) | 381^a^ | < 0.001 |
| Spectral Amplitude 452Hz_Asection | 1.680 (0.208) | 6.67 (3.62) | -4.156 | 0.001 |
| Spectral Amplitude 678Hz_Osection | 0.626 (0.070) | 1.405 (0.250) | 319^a^ | 0.001 |
| Spectral Amplitude 678Hz_Asection | 0.818 (0.095) | 3.824 (0.550) | -5.383 | < 0.001 |
| SNR 452Hz_Osection | 1.386 (0.598) | 5.360 (0.312) | 361^a^ | < 0.001 |
| SNR 452Hz_Asection | -0.066 (0.749) | 3.911 (0.743) | 324^a^ | < 0.001 |
| SNR 678Hz_Osection | -2.334 (0.894) | -0.443 (1.195) | 243^a^ | 0.212 |
| SNR 678Hz_Asection | -0.316 (0.891) | 5.137 (0.336) | 365^a^ | < 0.001 |

Results are expressed as mean (SD). ^a^ Mann-Whitney U test.

**Supplementary Table 7**

**Suppl. Table 7. Analysis by excluding extreme cases.** Main effects and interactions analyses from FFR_TFS_ derived parameters.

| Measure (N = 43) | Contrast Statistic | *p* |
| --- | --- | --- |
| **SNR_452 Hz** |  |  |
| Main effect GROUP | 31.881 | < 0.001 |
| Main effect STIMSECTION | 2.945 | 0.094 |
| Interaction | 0.000 | 0.998 |
| **SNR 678 Hz** |  |  |
| Main effect GROUP | 13.858 | 0.001 |
| Main effect STIMSECTION | 11.896 | 0.001 |
| Interaction | 2.616 | 0.113 |

Group = Newborns; Adults. StimSection = /o/ section; /a/ steady section.
